# Beyond Adaptive Immunity: Trained Innate Immune Responses as a Novel Frontier in Hepatocellular Carcinoma Therapy

**DOI:** 10.3390/cancers17071250

**Published:** 2025-04-07

**Authors:** Ching-Hua Hsieh, Pei-Chin Chuang, Yueh-Wei Liu

**Affiliations:** 1 Department of Plastic Surgery, Kaohsiung Chang Gung Memorial Hospital and Chang Gung University College of Medicine, Kaohsiung 83301, Taiwan; m93chinghua@gmail.com; 2 Department of Medical Research, Kaohsiung Chang Gung Memorial Hospital, Kaohsiung 83301, Taiwan; stemcorelab@cgmh.org.tw; 3 Department of General Surgery, Kaohsiung Chang Gung Memorial Hospital and Chang Gung University College of Medicine, Kaohsiung 83301, Taiwan; 4 Department of General Surgery, Kaohsiung Municipal Fong Shan Hospital—Under the Management of Chang Gung Medical Foundation, Kaohsiung 83091, Taiwan

**Keywords:** trained immunity, hepatocellular carcinoma (HCC), tumor microenvironment (TME), innate immune reprogramming, immune checkpoint inhibitors (ICIs)

## Abstract

Hepatocellular carcinoma (HCC) is a leading cause of cancer death globally, with the majority of cases detected at advanced stages when curative options are limited. Current systemic therapies, including immune checkpoint inhibitors, demonstrate limited efficacy with durable responses in only 15–20% of patients. This poor response is largely attributed to HCC’s immunosuppressive microenvironment, which blunts effective T-cell responses. By illustrating that innate immune cells can acquire memory-like characteristics through a process known as trained immunity, recent evidence has challenged the conventional belief that innate immunity is devoid of memory. This review investigates the potential of trained immunity, which is defined by the long-term functional reprogramming of innate immune cells through epigenetic, transcriptomic, and metabolic changes, to provide new therapeutic opportunities for HCC. We discuss mechanisms by which trained immunity can transform the HCC microenvironment, including enhanced inflammatory cytokine production, repolarization of tumor-associated macrophages toward anti-tumor phenotypes, increased immune cell infiltration, and improved bridging to adaptive immunity. We further evaluate emerging therapeutic strategies leveraging trained immunity principles, including BCG vaccination, β-glucan administration, cytokine-trained NK cell therapy, and innovative combination approaches. Finally, we address potential resistance mechanisms and future directions for clinical application. By integrating trained immunity into conventional immunotherapeutic regimens, we may significantly improve outcomes for HCC patients, potentially transforming advanced disease into a more manageable condition.

## Simple Summary

Hepatocellular carcinoma (HCC) is deadly and typically diagnosed when curative options are limited. Current immunotherapies help only 15–20% of patients due to the tumor’s immunosuppressive environment. This review explores how “trained immunity”—where innate immune cells develop memory-like properties after initial stimulation—offers a novel approach for HCC treatment. By reprogramming the immune environment through vaccines like BCG, compounds like β-glucan, or specialized cell therapies, this approach could overcome the limitations of current treatments. Combining trained immunity with conventional therapies may transform advanced HCC from a terminal diagnosis into a manageable condition by breaking through immunosuppressive barriers that typically block effective anti-tumor responses.

## 1. Introduction

Hepatocellular carcinoma (HCC) is a leading cause of cancer death globally, and it often develops in the presence of chronic liver inflammation. Most cases are diagnosed at stages too advanced for curative interventions. Current systemic therapies, including immune checkpoint inhibitors (ICIs), show limited efficacy with durable responses in only 15–20% of patients [1]. This failure to respond is related to HCC’s immunosuppressive milieu, which is abundant in tumor-associated macrophages, myeloid-derived suppressor cells, and regulatory T cells that hinder efficient immune responses [1,2]. This limited efficacy is largely attributed to the “cold” tumor immune microenvironment in HCC, which is enriched in immunosuppressive cells and factors that blunt adaptive T-cell responses. For instance, HCC tumors commonly harbor abundant tumor-associated macrophages (TAMs), myeloid-derived suppressor cells (MDSCs), and regulatory T cells, creating an immunosuppressive milieu that inhibits effector lymphocytes [3]. Overcoming this immunosuppressive barrier and transforming “cold” tumors into “hot” (T-cell-inflamed) tumors is a major therapeutic challenge [1].

The liver’s unique immunological environment, normally balanced between activation and tolerance, becomes disrupted in chronic disease, favoring tumor immune evasion [4]. Trained immunity offers a promising alternative approach. This phenomenon, where innate immune cells exhibit memory-like properties after initial stimulation, enables enhanced responses to subsequent challenges through epigenetic, transcriptomic, and metabolic reprogramming [2,5]. Unlike adaptive immunity, trained immunity provides broad-spectrum protection without antigen specificity—potentially advantageous against heterogeneous tumor antigens [2,6]. Trained immunity could rebalance this environment by enhancing innate cell responsiveness, potentially breaking immune tolerance even without strong adaptive immunity. Its antigen-agnostic nature might overcome tumor variants that escape T-cell recognition [2,7,8]. Early studies suggest trained immunity induction in HCC can activate both innate and adaptive responses, achieving tumor regression where conventional therapies fail [2].

This literature review will examine the role of trained immunity in HCC, with an emphasis on the underlying mechanisms, therapeutic strategies, modulation of the immune microenvironment, and resistance mechanisms. By integrating recent findings, we provide a comprehensive overview of how trained immunity could transform HCC therapy.

## 2. Mechanisms of Trained Immunity and Innate Immune Responses in HCC

### 2.1. Trained Innate Immunity: Definition and Core Mechanisms

Trained immunity represents the long-term functional reprogramming of innate immune cells following initial stimulation, resulting in enhanced responses to subsequent challenges [2]. This phenomenon occurs in myeloid cells, NK cells, and some non-immune cells [2,9]. Two key characteristics define trained immunity: it provides non-specific protection against unrelated secondary challenges, and it creates memory-like persistence in innate cells lasting weeks to months despite their lack of genomic recombination [2,10,11]. The foundation of trained immunity lies in epigenetic and metabolic reprogramming that alters baseline cellular activation [12].

Epigenetically, stimuli like β-glucan or Bacillus Calmette–Guérin (BCG) induce chromatin modifications that create a more “open” state at promoters of pro-inflammatory genes [2,13,14]. For instance, β-glucan-trained monocytes exhibit increased H3K4me3 at TNFα and IL6 promoters, enhancing transcription upon restimulation [15]. These changes, often reinforced by DNA methylation alterations, persist long-term [2]. Metabolically, trained cells upregulate aerobic glycolysis and cholesterol synthesis while downregulating oxidative phosphorylation [2,15]. This shift provides rapid energy and biosynthetic intermediates for robust responses. Importantly, the mTOR-HIF1α-mediated glycolytic switch in trained monocytes parallels cancer cell metabolism [2,16], warranting caution in therapeutic manipulation [2].

Various pattern recognition receptors trigger trained immunity. Classical inducers include β-glucans (via dectin-1) and BCG (via TLRs and NOD2) [2,17,18], which activate signaling cascades leading to epigenetic and metabolic reprogramming. Cytokine combinations can also induce training; IL-12/15/18 drives NK cells into a memory-like state with enhanced IFN-γ production and cytotoxicity against tumor cells [2]. These trained NK cells and monocytes can persist for extended periods despite their innate nature [19].

Critically, trained immunity is not uniformly pro-inflammatory; outcomes depend on stimulus type and context. While β-glucan and BCG typically induce pro-inflammatory states with increased TNFα, IL-6, and IL-1β production [15], other stimuli like chronic LPS exposure can cause tolerance with dampened responses. The balance between training and tolerance depends on factors including stimulus dose, duration, and cellular environment. Metabolically, robust mTOR and glycolysis activation favors training, while sustained activation of negative regulators like AMPK may induce tolerance [20]. IL-1β signaling drives trained immunity [2], while anti-inflammatory cytokines like IL-10 counteract training and promote tolerance [21].

In summary, trained immunity endows innate cells with a memory-like ability to mount stronger responses through enduring epigenetic and metabolic rewiring. This phenomenon broadens the host defense armamentarium beyond classical adaptive immunity. The next sections discuss how these mechanisms play out in the context of HCC, an inflammation-driven cancer where the innate immune system can be both a culprit in disease progression and a target for therapy.

### 2.2. Innate Immune Landscape in HCC: Baseline Immune Dysfunction and Tolerance

HCC develops in a chronically inflamed liver, yet paradoxically the tumor microenvironment (TME) is usually immunosuppressive or “tolerant”. Innate immune cells are prevalent in the liver and tumor stroma—including resident Kupffer cells (liver macrophages), infiltrating monocyte-derived macrophages, dendritic cells, NK cells, neutrophils, and innate-like lymphocytes. In a healthy liver, these cells maintain tolerance to constant gut-derived antigens while remaining able to combat infections. During chronic liver injury (due to hepatitis B or C, alcohol, NASH, etc.), this balance skews: innate cells become chronically activated but also exhausted or polarized toward pro-tumorigenic functions [2]. By the time HCC arises, the immune microenvironment is often characterized by the following:

Tumor-Associated Macrophages (TAMs): Macrophages are one of the most abundant immune infiltrates in HCC. They are frequently skewed to an M2-like, immunosuppressive phenotype by tumor-derived signals. TAMs produce IL-10, TGF-β and angiogenic factors, and express inhibitory checkpoints (e.g., PD-L1), thereby suppressing cytotoxic T-cell and NK-cell activity [3]. High densities of M2-polarized TAMs correlate with poor prognosis in HCC, whereas a higher ratio of pro-inflammatory M1 macrophages is associated with better outcomes [3,22]. TAMs are actively recruited and conditioned by the tumor: cancer cells and stromal cells, like cancer-associated fibroblasts, secrete CCL2 and colony stimulating factors that recruit monocytes and induce their differentiation into MDSCs and M2 TAMs via STAT3-activating cytokines (IL-6, etc.) [3]. The resultant TAMs are a key pillar of the “refractory” TME in HCC [3,22].

Myeloid-Derived Suppressor Cells (MDSCs): These immature myeloid cells are expanded in many cancers including HCC. MDSCs in HCC can arise from chronic inflammation-driven emergency myelopoiesis. They inhibit T-cell proliferation and NK function through arginase, inducible nitric oxide synthase (iNOS), and TGF-β/IL-10 production [23]. Tumor-secreted VEGF and other factors not only create blood vessels but also mobilize MDSCs and TAMs to the tumor site [1,23]. In HCC patients, elevated MDSC levels correlate with worse response to immunotherapy and shorter survival [1]. MDSCs further perpetuate immunosuppression by secreting VEGF and IL-10 themselves, creating a feedback loop [1,23].

Natural Killer (NK) Cells: NK cells are innate lymphocytes critical for surveilling and eliminating tumor cells, especially those with low MHC class I expression. In HCC, NK cells often display an exhausted or dysfunctional phenotype. Chronic exposure to TGF-β (abundant in cirrhotic and HCC tissues) downregulates NK activating receptors and cytotoxic mediators. Many HCCs also upregulate ligands like PD-L1 or HLA-E (the ligand for NK inhibitory receptor NKG2A) to directly inhibit NK activity [24]. Though dysfunctional, the presence of NK cells still counts: higher NK-cell infiltration in HCC tumors is linked to much better survival [25,26]. A meta-analysis of 26 studies found that patients with high intratumoral NK-cell counts had improved overall and disease-free survival (HR ~0.70 and 0.61, respectively) [25]. Thus, restoring NK-cell activity is a key goal for immunotherapy.

Neutrophils: Tumor-associated neutrophils (TANs) can have dual roles, but in HCC, a pro-tumor “N2” phenotype is common. Neutrophils are recruited by IL-8 (CXCL8) and other chemokines produced by HCC and stromal cells [27]. Once in the tumor, they can secrete neutrophil elastase, ROS, and growth factors that promote invasion and angiogenesis. Neutrophils also suppress adaptive immunity by secreting arginase and inducing T-cell apoptosis. In NASH-associated HCC, recent studies highlight neutrophils as major mediators of ICI resistance: fatty liver disease drives the accumulation of immature neutrophils that inhibit effective anti-tumor immunity [28]. For example, in murine NASH-HCC models unresponsive to anti-PD-1 therapy, co-treatment with a CXCR2 inhibitor (to prevent neutrophil recruitment) restored T-cell infiltration and tumor control [28,29]. This underscores how chronic inflammatory conditions alter neutrophil biology in ways that can hinder immunotherapy.

Dendritic Cells (DCs): Conventional DCs in HCC are often functionally impaired [30]. High VEGF levels in the HCC microenvironment interfere with DC differentiation and antigen presentation [1]. As a result, DCs in HCC may have reduced capacity to prime anti-tumor T cells [30]. Certain subsets like CD103^+ DCs are important for cross-presenting tumor antigens and recruiting NK/T cells, but their numbers are low in many HCC tumors. There is evidence that relieving immunosuppression (e.g., blocking CD47 on tumor cells) can activate intra-tumoral DCs and crosstalk with NK cells [1], suggesting that innate checkpoints on DCs/macrophages are manipulable to improve DC function.

Other Innate-like Cells: The liver contains NKT cells and γδ T cells, which straddle innate and adaptive immunity. These cells can respond rapidly to stress ligands on tumor cells. However, like NK cells, they are subject to tumor-induced inhibition. For instance, chronic lipid accumulation in NASH can activate NKT cells to produce cytokines that paradoxically exacerbate fibrosis and oncogenesis rather than mediate tumor rejection (a phenomenon termed “autoaggressive” NASH immunopathology) [31]. Innate lymphoid cells (ILCs) in the liver (ILC1, ILC2, ILC3 subsets) are less well studied in HCC, but ILC2 and ILC3 may contribute to the immunosuppressive cytokine milieu (IL-13, IL-22) that supports tumor growth [32,33].

**Figure 1:**
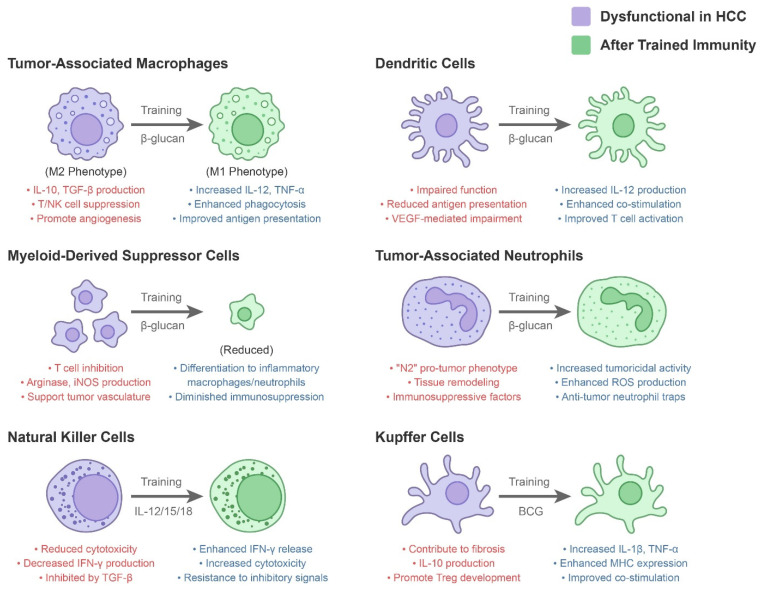
Key innate immune components in hepatocellular carcinoma and modulation by trained immunity.

The key innate immune components in HCC are listed in Table 1 and illustrated in Figure 1. The innate immune landscape in HCC typically favors tolerance and tumor promotion rather than suppression. Chronic inflammation from viral hepatitis or fatty liver disease continuously activates innate cells, driving them toward dysfunctional or immunoregulatory states. This persistent inflammation induces immunosuppressive factors (TGF-β, IL-10, IL-6, VEGF) from immune and non-immune cells [15], which promote wound-healing and fibrosis while impairing anti-tumor immunity. TGF-β and IL-10 inhibit NK and T-cell cytotoxicity, while VEGF not only creates vasculature but also recruits immunosuppressive cells and impairs DC maturation [1]. This environment challenges therapies that solely reinvigorate T cells, like immune checkpoint inhibitors. However, it presents an opportunity: reprogramming the innate compartment through trained immunity could reverse immunosuppression, potentially creating an “alert” immune state that breaks tolerance and provides a foundation for adaptive immune therapies.

## 3. Impact of Trained Immunity on the HCC Immune Microenvironment

Reprogramming the innate immune compartment through trained immunity has the potential to override the default immunosuppressive program in HCC. By inducing a more vigilant and pro-inflammatory state, trained immunity can modulate the TME in several beneficial ways:

Enhanced Inflammatory Cytokine Production: Trained monocytes/macrophages produce higher levels of IL-1β, TNF-α, IL-6, and chemokines upon stimulation [15]. In HCC, this counteracts the immunosuppressive IL-10/TGF-β environment. Increased IL-1 and TNF mature dendritic cells and activate endothelial cells, facilitating immune cell infiltration [34]. Heightened IL-12 promotes Th1 polarization and activates NK and CD8+ T cells [35].

Repolarization of Macrophages: Trained immunity can convert M2-like tumor macrophages to an M1 state. D-lactate delivery to HCC TAMs via nanoparticles effectively switched polarization from M2 to M1, downregulating M2 genes (Arg1, IL10) and upregulating M1 genes (IL12, iNOS, TNF-α) [36]. This reprogramming reduced immunosuppression and, when paired with anti-CD47 antibody, greatly prolonged survival in HCC-bearing mice [36,37].

Increased Immune Cell Infiltration: Trained immunity transforms “cold” tumors to “hot” by enabling T- and NK-cell infiltration [38]. Trained macrophages/dendritic cells produce CXCL9 and CXCL10, attracting CXCR3+ T cells 1. CD47 blockade increased such chemokines and established a CD103+ DC–NK cell axis, bringing more NK and T cells into tumors [1]. Similarly, β-glucan administration turned “cold” tumors “hot” in pancreatic models, a principle potentially applicable to HCC [2].

Direct Anti-tumor Effects: Trained innate cells show enhanced cytotoxicity. Trained neutrophils increase ROS production and extracellular traps that kill tumor cells [2,39]. Trained macrophages demonstrate superior phagocytic capacity [39]. Cytokine-trained NK cells show heightened killing activity in various cancer models including HCC 2. Adoptive transfer of IL-12/15/18-trained NK cells showed superior antitumor effects in HCC models [2].

Bridging to Adaptive Immunity: Trained immunity potentiates adaptive responses by activating APCs and providing pro-inflammatory context for better T-cell priming [40]. BCG not only activates innate cells but requires T cells for full anti-tumor effect in HCC models [2]. A single BCG injection led to robust T-cell recruitment alongside macrophages, and T cell blocking abrogated BCG-induced tumor regression [2]. This creates a virtuous cycle: innate training supports T-cell responses through cytokines and improved antigen presentation, while T cells execute tumor killing and provide IFN-γ that further activates macrophages and NK cells [41].

By generating an immunostimulatory loop between innate and adaptive cells, trained immunity creates a multiplier effect on anti-tumor immunity in HCC. This contrasts sharply with the basal HCC tumor microenvironment dominated by immunosuppressive loops. Trained immunity interventions essentially “reset” the innate immune tone from tolerogenic to active, breaking tumor-driven immune silencing. However, it is crucial to recognize the paradoxical nature of pro-inflammatory environments: persistent or severe inflammation may foster genomic instability and tumor proliferation long-term [15]. Since persistent inflammation in cirrhosis predisposes to HCC development, therapeutic induction of trained immunity must be carefully controlled and likely time-limited—sufficient to provoke anti-tumor immune responses without causing undue tissue damage or fueling paraneoplastic inflammation. These considerations will be explored further in sections addressing resistance mechanisms and future directions.

Having discussed how trained immunity can mechanistically reshape immune responses in HCC, we now turn to the clinical realm. How can these insights be translated into therapy? What strategies have been tested or proposed to induce trained immunity in HCC patients, and what are their outcomes or promises? The next section delves into therapeutic strategies, from repurposing old vaccines like BCG to developing novel trained immunity activators and examines how immune modulation via these interventions could improve HCC treatment.

## 4. Clinical Implications

### 4.1. Trained Immunity-Based Therapeutic Strategies in HCC

The concept of manipulating trained immunity for cancer therapy is gaining momentum, and HCC is a prime candidate given its immunosuppressive milieu and partial resistance to conventional immunotherapies. Several therapeutic strategies have emerged that aim to “train” the immune system to better recognize and eliminate HCC. These approaches draw on agents that can induce trained immunity, often repurposed from infectious disease contexts, as well as cellular therapies that leverage innate immune memory. Below, we review the key potential therapies related to trained immunity in HCC, summarizing them in Table 2 with their mechanisms and current status.

Bacillus Calmette–Guérin (BCG) Vaccine: BCG, originally developed for tuberculosis, induces trained immunity and has been successfully used for bladder cancer [47,48]. Preclinical HCC studies are promising: a single subcutaneous BCG dose significantly reduced tumor burden and extended survival in orthotopic mouse models, outperforming anti-PD-1 therapy. BCG increased tumor infiltration by T cells and M1 macrophages while elevating IFN-γ levels [2]. Its efficacy requires both adaptive and trained innate immunity, as blocking either pathway eliminated anti-tumor effects [2]. Mechanistically, BCG engages NOD2 and TLRs on monocytes/macrophages to induce training and stimulates emergency myelopoiesis, producing tumoricidal neutrophils and monocytes [2]. A case report documented disease control in liver metastatic neuroendocrine carcinoma using combined BCG and PD-1 inhibition [2]. BCG’s established safety profile and FDA approval for bladder cancer provides a strong rationale for HCC repurposing, particularly as an adjuvant after tumor ablation or resection. Its ability to reduce liver fibrosis could benefit cirrhotic patients [49]. The current research explores optimal administration routes: intravenous delivery may induce stronger training by seeding bone marrow, while intralesional or intraperitoneal administration could focus effects in the liver [2]. Though large clinical trials remain pending, BCG represents a promising, cost-effective trained immunity therapy for HCC [48,49]

β-Glucan and Yeast-Derived Particulates: β-glucans from fungal cell walls are established training agents for monocytes [50,51]. These compounds have been studied as immunomodulators in cancer models and clinical trials for colorectal and other cancers [52,53]. For HCC, both oral administration (training gut-associated macrophages and Kupffer cells) and particulate β-glucan injections targeting the liver show promise. While direct HCC clinical data are limited, insights from other solid tumors are relevant. In pancreatic cancer models, oral β-glucan improved post-surgical survival by inducing trained monocytes that produced more TNF-α and attracted T cells, functioning independently of adaptive immunity [2]. Intraperitoneal particulate β-glucan (whole glucan particles (WGPs)) homes to the liver and spleen, polarizing macrophages toward a tumoricidal state and controlling metastases in liver metastatic disease models [2]. Mechanistically, β-glucan engages the dectin-1 receptor on myeloid cells, triggering a cascade via Syk kinase and NLRP3 inflammasome that leads to IL-1β production, a key training cytokine [54]. An innovative approach uses β-glucan particles as delivery vehicles, loading tumor antigen or anti-cancer drugs into hollow β-glucan shells for uptake by phagocytes, simultaneously delivering payloads and inducing trained immunity [2]. Despite no HCC patient trials yet, a phase I study could evaluate oral yeast β-glucan as an adjunct to checkpoint inhibitors or loco-regional therapies. Preclinical evidence suggests β-glucan might synergize with PD-1/PD-L1 inhibitors by transforming “cold” tumors to “hot”—combining β-glucan with anti-PD-L1 therapy significantly extended survival in murine pancreatic cancer [49]. A similar combination could potentially increase ICI response rates in HCC by pre-conditioning patients with β-glucan to enhance myeloid cell activity.

Cytokine-Trained NK Cell Therapy: NK cells can be “trained” through pre-activation with cytokines including IL-12, IL-15, and IL-18 [55]. A clinically viable approach involves the ex vivo priming of autologous NK cells with these cytokines before patient infusion. Clinical trials in leukemia and solid tumors have demonstrated the safety and efficacy of adoptively transferred cytokine-induced memory-like NK cells [50]. HCC, typically liver-confined, is particularly suitable for regional or systemic NK cell therapy. Early-phase HCC trials of NK infusions have shown tumor shrinkage or slowed progression [43], with incorporating trained NK protocols as the logical next step. Preclinical studies demonstrated improved tumor control in HCC-bearing mice infused with IL-12/15/18-activated NK cells, particularly in combination therapies [52]. IL-15 superagonists like N-803-modified IL-15 potently stimulates NK and CD8 T cells, showing promise in other cancers, including bladder cancer when combined with BCG [2,42]. IL-15 agonists in HCC could enhance NK cell numbers and activity within the liver, with ongoing clinical trials exploring their combination with checkpoint inhibitors in advanced liver cancers [56,57]. CAR-NK cells targeting HCC-specific antigens like glypican-3 represent another frontier, with cytokine training potentially enhancing persistence and efficacy [2].

Toll-Like Receptor (TLR) Agonists and Other Innate Adjuvants: A variety of TLR agonists (synthetic or microbial derivatives) can activate innate immune cells and have been tested as cancer adjuvant therapies. In HCC, one example is CpG oligodeoxynucleotide (TLR9 agonist), which has been combined with other treatments. The rationale is that activating liver-resident dendritic cells and macrophages via TLR9 will promote tumor antigen presentation and T-cell recruitment [58,59]. TLR7/8 agonists (like resiquimod) could similarly be used to activate Kupffer cells and monocytes in the liver [44]. One challenge is that TLR agonists can also upregulate checkpoint molecules on myeloid cells—for instance, TLR stimulation can induce PD-L1 expression on macrophages [15]. This suggests that TLR agonists might best be used in combination with ICIs to prevent induced checkpoints from negating their immune-boosting effects. Another innate adjuvant route is the STING agonists, which activate the cytosolic DNA-sensing pathway, leading to a strong Type I IFN response that can mature dendritic cells and induce T cell-attracting chemokines [60,61]. The intratumoral injection of STING agonists in liver tumor models has shown potent immune activation and synergy with checkpoint inhibitors [1,62]. Though not a classic “trained immunity” (STING effect is more acute), repeated low-dose STING stimulation might imprint a sustained myeloid activation in the liver. Ongoing trials in solid tumors for STING agonists could pave the way for HCC applications.

Trained Immunity-Based Vaccines or Cell Therapies: Beyond single agents, researchers are exploring vaccination strategies that incorporate trained immunity principles. One idea is to use BCG or other live attenuated microbes as adjuvants for HCC tumor-antigen vaccines. By co-delivering tumor antigens (like peptides from glypican-3, an HCC antigen) with a potent trainer like BCG or a synthetic β-glucan conjugate, one could stimulate both innate training and adaptive specific immunity [45,46]. Preclinical studies have shown that linking tumor antigens to β-glucan particles leads to better T-cell responses, presumably because the antigen is taken up by trained APCs that provide stronger co-stimulation [2]. Another concept is the adoptive transfer of trained monocytes [63,64]. Monocyte transfusions have been attempted in sepsis; in cancer, one could isolate a patient’s monocytes, train them in vitro with a cocktail (like β-glucan and cytokines), then reinfuse them so they home to the tumor and liver. These cells would carry an epigenetic memory of training and could secrete high levels of TNFα/IL-1 upon encountering the tumor. While this is still hypothetical for HCC, it represents a personalized cell therapy angle for trained immunity.

Combination Therapies with Standard HCC Treatments: Trained immunity inducers can also be combined with existing HCC therapies for potential additive or synergistic effects. For example, lenvatinib and sorafenib served as first-line therapy of advanced hepatocellular carcinoma [65]. The anti-angiogenic drug lenvatinib normalizes tumor vasculature and was shown to increase immune cell infiltration in HCC [1]. Sorafenib, a multikinase inhibitor, downregulates key cellular activities, such as those related to the mitochondrial metabolism and the collagen synthesis, and upregulates pathways associated with the adaptation and survival of cells [66]. Combining an anti-angiogenic with a trained immunity stimulus might yield better immune infiltration than either alone, e.g., bevacizumab could improve access of trained myeloid cells to the tumor [67]. Additionally, radiofrequency ablation or transarterial chemoembolization cause tumor cell death and release of antigens; administering a trained immunity adjuvant around the time of these procedures could convert the death of immune cells into a systemic anti-tumor immune response. A small pilot in HCC found that injecting a TLR3 agonist (poly-ICLC) into the tumor ablation zone increased T-cell activation afterwards [1]. This aligns with the concept of using innate stimuli to turn local therapy into an in situ vaccine.

When implementing these therapies, careful attention to the immune modulation of the tumor microenvironment is essential. While aiming for potent anti-tumor immune responses, we must anticipate how tumors might adapt to or resist these interventions. Rational combinations and optimal scheduling of treatments (such as whether to administer trained immunity inducers before or after checkpoint inhibitors) require careful consideration [2]. The next section examines how trained immunity reshapes the HCC immune environment, potential tumor resistance mechanisms, and strategies to overcome these challenges.

### 4.2. Immune Modulation of the HCC Microenvironment by Trained Immunity

A central goal of applying trained immunity in HCC therapy is to modulate the TME from an immune-suppressive state to an immune-active one. Trained immunity-based interventions seek to tip the scales by introducing a pro-inflammatory bias and enhancing immune cell recruitment and function in the tumor. Here, we highlight key aspects of this immune modulation and how they manifest in HCC:

Converting “Cold” Tumors to “Hot”: Many HCC lesions are “immune desert” or “immune excluded” phenotypes, meaning few T cells are present or they are stuck at the margins of the tumor. Successful immune modulation is often visible as an increase in intratumoral CD8^+ T cells and Th1 cells (a “hot” tumor) [68]. For instance, after BCG treatment in HCC models, previously poorly infiltrated tumors became populated with CD8^+ T cells throughout, coinciding with tumor regression [2,49]. Clinically, the combination of anti–PD-L1 atezolizumab with anti-VEGF bevacizumab has already shown that modulating the microenvironment (by normalizing vasculature and reducing myeloid suppression via VEGF blockade) can raise the effectiveness of T-cell checkpoint therapy in HCC [1]. Trained immunity inducers can further this conversion. By activating endothelial cells and secreting chemokines, they ensure effector cells enter the tumor core rather than remain at the periphery. Measuring pre- and post-therapy biopsies for T-cell density and localization is one way of gauging the immune modulation achieved by a trained immunity therapy.

Augmenting the Type 1 Immune Response: HCC progression is often fueled by Type 2 (Th2) or regulatory immune responses—high IL-4, IL-5, IL-13 from Th2 cells, and IL-10, TGF-β from regulatory T cells (Tregs) and M2 macrophages [69]. A shift to a Type 1 response (dominated by IFN-γ, IL-2, IL-12) is desirable for anti-tumor activity. Trained immunity skews toward Type 1. For example, trained macrophages produce more IL-12 and less IL-10 [36], favoring Th1 and cytotoxic T-cell responses. Trained NK cells secrete abundant IFN-γ [2], which not only directly inhibits tumor proliferation but also activates M1 macrophages and DCs (creating a positive feedback loop). There is evidence that patients whose HCC tumors have an “immune active” transcriptomic signature (high IFNG, T-bet, etc.) respond better to immunotherapy [1,70,71]. Thus, therapies that induce a trained immunity profile could turn on these Type 1 signatures in initially “cold” HCCs, potentially making them respond to ICIs where they previously would not. In essence, trained immunity provides the cytokine milieu that ICIs need to work—you need IL-12 and IFN-γ to make T cells function once PD-1 is lifted [72,73].

Breaking Immune Tolerance in the Liver: The liver normally maintains immunological tolerance to food antigens and commensals, which HCC exploits for immune evasion. Trained immunity can transiently disrupt this tolerance, though a careful balance must be maintained to avoid autoimmunity or hepatitis. Short-term, localized tolerance disruption can be advantageous—for example, intratumoral TLR agonist injection can induce localized inflammation that temporarily overcomes tolerance, exposing the tumor to immune attack without triggering systemic autoimmunity. This tolerance-breaking approach is exemplified by combining checkpoint inhibition with trained immunity: while checkpoint inhibitors release T-cell inhibition, the inherently tolerogenic liver environment may still prevent T-cell activation. Adding a trained immunity stimulus, such as a hepatic artery infusion of a TLR9 agonist, provides an adjuvant effect that signals danger, stimulating enhanced immune responses. Preliminary results from a study combining TLR9 agonist SD-101 with pembrolizumab showed increased chemokine levels and tumor responses, indicating partially overcome tolerance with manageable hepatotoxicity [74].

Mitigating Immunosuppressive Factors: Trained immunity can indirectly reduce levels of immunosuppressive cytokines and cells. For example, there is crosstalk where a robust IL-1/IL-12/IFN-γ environment downregulates the influence of IL-10 and TGF-β (which drive TAMs and Tregs) [75]. Moreover, an influx of trained monocytes might outcompete MDSCs in the tumor for growth factors and space, effectively reducing MDSC presence [76]. Some trained immunity inducers lead to apoptosis of suppressive cells—high-dose CpG, for instance, can cause tolerogenic plasmacytoid DCs to undergo apoptosis while activating conventional DCs [77]. Trained immunity inducers like BCG also have anti-angiogenic effects indirectly (through cytokines like IFN-γ, which can normalize vasculature). A more oxygenated, less hypoxic tumor after such modulation is also more susceptible to immune attack, because hypoxia itself is immunosuppressive (induces checkpoints like PD-L1 and enzymes like adenosine). By modulating the microenvironment to be more inflammatory and less hypoxic, as observed in BCG-treated mouse livers which had metabolic pathway shifts [49], trained immunity could help overcome these microenvironmental barriers.

Microbiota Manipulation and HCC Immunotherapy: The gut microbiome critically shapes systemic and anti-tumor immunity, including immunotherapy responses. Dysbiosis can drive chronic liver inflammation and immune dysfunction, while a diverse microbiota supports anti-tumor activity. Recent evidence has suggested that gut microbial composition may predict HCC immunotherapy outcomes. A study in unresectable HCC found significant differences in baseline fecal microbiota between anti-PD-1 responders and non-responders, with the enrichment of *Lachnospiraceae* and *Veillonella* in responders versus *Prevotella* in non-responders. Responders also had higher levels of certain bile acids (e.g., ursodeoxycholic acid), highlighting the connection between microbiota metabolism and checkpoint inhibitor efficacy [78]. These findings indicate the gut microbiome can modulate the HCC immune microenvironment, suggesting potential for microbiota-targeted therapeutic strategies.

Microbiota-modifying interventions have shown promise for enhancing immunotherapy efficacy and safety in various cancers. In humans, fecal microbiota transplantation from immunotherapy-responsive donors has induced clinical responses in PD-1-refractory melanoma patients, with increased tumor immune cell infiltration. Though experimental in HCC, these approaches demonstrate the potential of microbiota manipulation (via diet, probiotics, or FMT) to create a more immunotherapy-responsive environment. Additionally, a healthy gut microbiome may reduce immunotherapy toxicity, as certain commensal bacteria (e.g., *Bacteroides vulgatus* and *Faecalibacterium prausnitzii*) correlate with a lower risk of immune-related colitis during checkpoint inhibitor treatment. Thus, microbiome modulation could enhance both the efficacy and safety of HCC immunotherapies, an area of growing research interest.

Synergizing with Other Treatments: Immune modulation via trained immunity does not occur in isolation; it ideally works in synergy with other therapies. For example, radiotherapy or thermal ablation kills tumor cells, but a trained immune system will more effectively clean up the debris and present tumor antigens to T cells, turning local therapy into systemic immunity (the abscopal effect) [79,80]. Indeed, in preclinical studies, mice pre-treated with a trained immunity inducer and then given tumor irradiation had better tumor control than those given irradiation alone [15]. Another synergy is with metabolic therapies: some HCCs have high lactate production which polarizes TAMs to M2 [81]. Interventions like the D-lactate nanoparticle we discussed not only reprogram TAMs but also alter the metabolic microenvironment (by using up lactate or blocking its signaling) [36]. This makes the tumor a less hospitable place for cancer cells that rely on an M2-rich, immunosuppressed niche.

Effective trained immunity modulation in HCC creates an environment where immune cells dominate cancer cells, inverting the typical advanced HCC scenario. This transformation manifests as increased immune infiltration, elevated pro-inflammatory cytokines, normalized vasculature, and fewer MDSCs and Tregs. Clinically, tumors may shrink or become responsive to previously ineffective immunotherapies. Success markers include transient serum cytokine elevations (IL-6, IL-1β), improved tumor CD8+/Treg ratios, and conversion from non-inflamed to inflamed gene signatures in biopsies.

However, tumors are not passive bystanders in this process. HCC can adapt and develop resistance mechanisms to evade the reinvigorated immune attack. In the next section, we address these resistance mechanisms, which represent the flip side of the coin: as we push the immune system to fight the cancer, how might the cancer push back, and what strategies can counteract that dynamic?

## 5. Resistance Mechanisms and Immune Evasion in the Context of Trained Immunity

Cancer immunoediting is a continuous battle—as the immune system exerts pressure, the tumor cells may adapt to survive. When we introduce trained immunity-based therapies in HCC, we heighten immune pressure on the tumor. This can lead to improved tumor control, but it can also select for tumor escape variants or induce counter-regulatory mechanisms. Understanding these resistance pathways is crucial to refining therapeutic strategies. Key potential resistance and evasion mechanisms in HCC include the following:

Upregulation of Immune Checkpoints and Inhibitory Molecules: In response to an activated immune environment, tumor cells and immune cells can increase the expression of inhibitory ligands that dampen the immune attack. For instance, IFN-γ (produced abundantly when trained immunity activates NK and T cells) can induce PD-L1 expression on tumor cells and macrophages [82,83]. Thus, an HCC that is under assault might quickly upregulate PD-L1, PD-L2, or other checkpoints (like HLA-E to engage NKG2A on NK cells, or CD274 which encodes PD-L1) as a shield [2]. This was observed in some studies where TLR agonist treatment of macrophages led to increased PD-L1 along with the desired activation, necessitating PD-1 blockade to fully realize the anti-tumor effect [15]. Likewise, HCC cells might increase the expression of CD47 (the “don’t eat me” signal) in the face of aggressive macrophages, to avoid phagocytosis [1]. These findings suggest that combination therapies are needed, e.g., if using a TLR agonist or BCG, one might pre-emptively combine it with anti-PD-1 or anti-CD47 to block the predictable counterattack of the tumor (PD-L1, CD47 upregulation). Notably, an ongoing HCC trial adds anti-CD47 to tremelimumab + durvalumab (CTLA-4 and PD-L1 blockers) for this reason.

Emergence of “Immune-Evasive” Tumor Cell Clones: HCC is heterogeneous. Under immune pressure, variants that are less immunogenic can be selected. For example, clones with reduced expression of antigen presentation molecules (MHC class I loss or alteration) may gain a growth advantage if cytotoxic T cells are active [84]. While trained immunity relies less on specific antigen recognition, there is still some dependency: NK cells target cells with low MHC class I, but if an HCC clone manages to both lose neoantigen expression (to evade T cells) and retain just enough class I to avoid NK cells, it could theoretically slip through [85,86]. Another adaptation is alteration in death pathways—tumor cells might upregulate survival signals or anti-apoptotic proteins (like Bcl-2, IAPs) to resist the cytotoxic effects of NK and T cells. There is evidence that chronic exposure to IFN or TNF can make tumor cells more resilient to those cytokines over time [87]. Thus, a trained immunity onslaught could inadvertently select for tumor cells that are cytokine-resistant or insensitive to macrophage/NK-induced death signals. Monitoring tumor biopsy molecular changes during treatment can alert clinicians to such adaptations (e.g., loss of beta-2-microglobulin indicating MHC class I loss).

Immune Privilege via Physical Barriers: Tumors may reinforce physical and physiological barriers to immune cell infiltration. HCC can increase fibrosis or alter the tumor vasculature to impede immune cell entry even in the presence of strong chemokines. For example, excess collagen deposition could trap macrophages at the periphery. Some HCCs produce high levels of angiopoietin and other factors to create abnormal blood vessels that T cells/NK cells cannot penetrate [88]. Under immune pressure, a tumor might ramp up these processes, essentially walling itself off. This is where combining anti-fibrotic or anti-angiogenic drugs (like the anti-VEGFR2 antibody ramucirumab or anti-fibrotic agents) with trained immunity inducers could help. Indeed, anti-VEGF therapy has been shown to normalize vessels and allow T-cell penetration in HCC [1], so a tumor’s attempt to hide via vasculature can be countered by continuing bevacizumab in combination with immune activation.

Expansion of Regulatory Cell Populations: Tumors under attack can paradoxically stimulate the expansion of regulatory immune cells as negative feedback. In HCC, two populations to watch are Tregs and IL-10-producing regulatory macrophages. While trained immunity tends to diminish these, the immune system itself might try to self-regulate if inflammation is high. For instance, a surge of IL-1 and IFN-γ might lead to increased recruitment of Tregs (since Tregs express CXCR3 and can be drawn by IFN-induced chemokines just like effector T cells) [89]. These Tregs could then accumulate and secrete IL-10 or consume IL-2, dampening the effector functions. Similarly, certain monocytes in a highly inflammatory environment may develop into myeloid-derived suppressor cells as a homeostatic strategy. A study in melanoma patients found that persistent training stimuli caused a subgroup of monocytes to become suppressive to prevent autoimmunity [36]. We must consider that in a patient, chronic exposure to a trained immunity inducer could trigger feedback inhibition and hence, cyclic or limited dosing might be necessary. One strategy to handle this is the intermittent dosing of training agents and using low-dose cyclophosphamide or other Treg/MDSC-targeting drugs to selectively suppress the suppressors. In cancer therapy, low-dose cyclophosphamide has been used to reduce Tregs in the patient prior to giving a cancer vaccine [90].

Metabolic Adaptations and Nutrient Competition: A metabolic competition exists in the tumor microenvironment where activated immune cells require substantial nutrients while glycolytic tumor cells outcompete them for glucose, glutamine, and oxygen, effectively starving immune cells in poorly perfused areas [91]. Under immune pressure, HCC may enhance glucose uptake and lactate production, creating an immunosuppressive acidic environment that inhibits T and NK cells while promoting M2 macrophages [81]. Without addressing this metabolic landscape, tumors might exploit it by increasing immunosuppressive metabolites like lactic acid and adenosine. Adenosine signaling via A2A receptors suppresses immunity in HCC [92], and immune pressure can upregulate CD39/CD73 ectoenzymes that generate adenosine [93], potentially requiring targeted therapeutics [94]. Trained immunity partially counters these mechanisms: IFN-γ from trained NK cells inhibits angiogenesis, reducing tumor nutrient supply, while trained macrophages utilize alternative fuels, reducing glucose dependency [95]. Combining trained immunity with metabolic interventions like metformin or dichloroacetate, which restore NK function by inhibiting tumor lactate production [96], could overcome these metabolic resistance strategies.

Influence of Etiology (Viral vs. NASH) on Resistance: Emerging evidence indicates HCC etiology (HBV, HCV, NASH, alcohol) significantly impacts the immune microenvironment [97,98]. NASH-related HCC shows lower immunotherapy sensitivity, likely due to baseline abundance of immunosuppressive cells including neutrophils and exhausted CD8 T cells [28]. Applying trained immunity therapy in NASH-HCC patients may initially encounter greater resistance, as trained monocytes could be rapidly deactivated by elevated IL-10 and fatty acids in NASH livers, while trained neutrophils might revert under NASH-associated cytokines. In contrast, viral HCC presents different challenges. Ongoing viral antigens may partially train the immune system (acute viral hepatitis induces interferons and trained phenotypes), but chronic infection leads to immune exhaustion. In chronic HBV-HCC, the virus likely drives immunotolerance contributing to carcinogenesis, suggesting that effective treatment might require combining antiviral therapy with immune training.

Resistance mechanisms thus differ by HCC subtype: NASH-HCC primarily resists through neutrophil-mediated and metabolic suppression, while viral HCC employs high PD-L1 expression and exhaustion pathways. Tailoring adjunct treatments accordingly—such as CXCR2 inhibitors for NASH-HCC neutrophils [28] and antiviral therapy for HBV-HCC [99]—will be crucial for maximizing the effectiveness of trained immunity approaches.

## 6. Future Directions Section

The convergence of trained immunity and cancer immunotherapy in HCC remains in the early stages despite encouraging evidence. Several key research priorities should be pursued:

Optimizing Inducers and Combinations: Beyond BCG and β-glucan, novel TLR agonists, attenuated viruses, or endogenous alarmins may induce more potent trained immunity. Systematic comparisons could identify optimal regimens, while combination approaches (systemic and local inducers) might prove synergistic. The timing sequence with other therapies requires investigation—should training precede checkpoint inhibitors, or vice versa? Integration with current standards like atezolizumab plus bevacizumab warrants clinical evaluation.

Biomarkers and Patient Selection: Predictive biomarkers are needed to identify likely responders. Potential markers include baseline monocyte/NK cell function, tumor mutational landscape, and epigenetic signatures of trained immunity. Gene expression profiling might reveal an “innate immune activation signature” correlating with response, enabling personalized treatment selection.

Dosing, Timing, and Delivery Optimization: Refined protocols for dose, frequency, and administration route are essential. Excessive dosing may induce tolerance rather than training. Novel delivery systems using nanoparticles or hydrogels could enhance targeting and efficacy while minimizing systemic exposure, particularly important for liver-directed approaches.

Safety Management: Safety remains paramount, especially for cirrhotic HCC patients. Strategies include better spatial control through localized delivery, inducible systems activated only in tumor microenvironments, and robust monitoring protocols to detect excessive inflammation before clinical manifestation. Animal models mimicking patient conditions should verify safety before clinical implementation.

Personalized Approaches: HCC heterogeneity demands personalized strategies. Future approaches may involve the ex vivo testing of patient-derived samples to determine optimal treatments based on tumor “trainability”. Integrated therapies combining trained immunity inducers with metabolic supports and checkpoint inhibitors could address multiple tumor evasion mechanisms simultaneously. Prevention strategies for high-risk populations represent another promising frontier.

## 7. Conclusions

The integration of trained immunity into HCC therapy represents a paradigm shift—leveraging the ancient arm of the immune system to fight a modern scourge. The vision is a well-orchestrated immunotherapeutic regimen in HCC where innate and adaptive immunity are both engaged: the innate immune system provides a powerful, broad, and sustained first strike and supportive role, while the adaptive immune system executes precision killing of the cancer. If successful, this dual approach could substantially improve outcomes for HCC patients, increasing cure rates in early disease and turning advanced HCC into a more controllable chronic condition. As our understanding deepens, we may find that trained immunity is not only a therapy but also a prevention strategy, for instance, could high-risk cirrhotic patients receive a trained immunity booster (like BCG) to prevent HCC development by enhancing immunosurveillance? Initial epidemiological hints suggest patients who received BCG vaccination have lower cancer rates [2]. Such ideas blur the line between immunotherapy and immunoprevention. They exemplify the bold, innovative thinking that the field is now embracing.

## Figures and Tables

**Table 1:** Key innate immune components in HCC, their dysfunctional roles, and modulation by trained immunity.

Innate Immune Component	Dysfunctional Role in HCC Microenvironment	Modulation by Trained Immunity
Tumor-Associated Macrophages (TAMs)	Often M2-polarized; secrete IL-10, TGF-β, VEGF; suppress T/NK cells; promote angiogenesis and tumor growth [3]. High TAM burden correlates with poor prognosis [3].	Training (e.g., with BCG or β-glucan) can repolarize macrophages toward an M1 phenotype, increasing IL-12, TNF-α, and antigen presentation [34]. Trained macrophages show enhanced phagocytosis and ROS production against tumor cells, potentially converting an immunosuppressive milieu into an inflammatory one [2].
Myeloid-Derived Suppressor Cells (MDSCs)	Expanded in HCC; inhibit T-cell proliferation via arginase, iNOS, IL-10; support tumor vasculature and metastasis [23]. Present in blood and tumors of HCC patients, especially in advanced disease [23].	Certain trained immunity strategies aim to differentiate or deplete MDSCs. For example, β-glucan can drive myeloid precursors towards inflammatory macrophages and neutrophils, reducing the immunosuppressive MDSC pool [2]. Trained monocytes may also be resistant to tumor-derived suppressive signals, diminishing MDSC accumulation [2].
Natural Killer (NK) Cells	Key anti-tumor effectors, but often dysfunctional in HCC due to TGF-β and chronic stimulation. Show reduced cytotoxicity and IFN-γ production; some HCCs upregulate HLA-E or shed NKG2D ligands to evade NK attack. High NK infiltration predicts better survival [25,26].	Cytokine-induced trained NK cells have enhanced IFN-γ release and cytotoxicity upon encountering tumor cells [2]. IL-12/15/18 “memory-like” NK cells or IL-15 superagonist-expanded NK cells can overcome some inhibitory signals and more efficiently lyse HCC cells (even those with low MHC-I) [2]. Trained NK cells are being tested in adaptive cell therapy to improve tumor control in HCC [2].
Neutrophils (TANs)	Often exhibit an “N2” pro-tumor phenotype: secreting proteases, ROS that cause tissue remodeling, and suppressive factors. Neutrophil-to-lymphocyte ratio is a negative prognostic indicator in HCC [28,29].	Trained immunity (e.g., via β-glucan) can reprogram neutrophil production and function. β-glucan has been shown to induce trained granulopoiesis, yielding neutrophils with increased tumoricidal activity (via ROS and neutrophil extracellular traps) [2]. Trained neutrophils may be less prone to the immature, immunosuppressive phenotype seen in NASH-HCC [28,29]. However, excessive neutrophil activation must be balanced to avoid collateral damage.
Dendritic Cells (DCs)	Conventional DCs in HCC are often functionally impaired [30]. High VEGF levels in the HCC microenvironment interfere with DC differentiation and antigen presentation [1]. As a result, DCs in HCC may have reduced capacity to prime anti-tumor T cells [30].	Trained monocytes give rise to more potent DCs with increased IL-12 production and co-stimulatory molecule expression [2]. In a trained environment, DCs may overcome tumor-induced paralysis, leading to better activation of tumor-specific T cells. Some trained immunity adjuvants (like CpG DNA or LPS analogs) directly activate DCs to mature and migrate into lymph nodes, bridging innate and adaptive responses [2].
Kupffer Cells (Liver Resident Macrophages)	In chronic liver disease, Kupffer cells contribute to fibrosis and can become tolerant to endotoxin (reducing their cytokine output). They form part of the immunosuppressive stroma in HCC, producing IL-10 and promoting Treg development [2].	BCG or other inducers can potentially train Kupffer cells. Trained Kupffer cells would secrete more pro-inflammatory cytokines (IL-1β, TNF-α) upon sensing tumor antigens or danger signals, thereby activating other immune cells in the liver. In trained mice, liver macrophages have shown increased expression of MHC and co-stimulatory molecules [2,35], suggesting improved capacity to stimulate anti-tumor T cells locally.

**Table 2:** Potential therapies targeting trained immunity in HCC, categorized by mechanism.

Mechanism Category	Therapy	Mode of Action and Relevance to Trained Immunity in HCC
Trained Immunity Induction	BCG Vaccine	Engages pattern recognition receptors (NOD2, TLRs) on monocytes/macrophages, driving them into a trained state. In preclinical HCC models, a single BCG dose significantly reduced tumor burden and outperformed anti-PD-1 therapy [2].
	β-Glucan (yeast-derived)	Binds dectin-1 on myeloid cells, triggering Syk–NLRP3 inflammasome signaling and IL-1β release, a key trained immunity mechanism. β-Glucan-trained macrophages and neutrophils have increased tumoricidal activity [2]. Synergized with PD-1 blockade in murine models [2].
Cytokine-Based Therapy	IL-15 Superagonist (N-803)	Expands and activates NK and CD8^+ T cells in vivo. Being tested in clinical trials for solid tumors, including liver cancer [42].
Adoptive Cell Therapy	Cytokine-Trained NK Cells	IL-12/15/18-trained NK cells exhibit enhanced IFN-γ secretion and cytotoxicity against HCC cells [2]. Early-phase trials for NK cell therapy in HCC are ongoing [43].
Immune Checkpoint Modulation	Anti-NKG2A and Anti-CD47	Anti-NKG2A prevents HCC cells from engaging NK-cell inhibitory receptors, enhancing NK cytotoxicity [25]. Anti-CD47 removes the “don’t eat me” signal, enabling trained macrophage phagocytosis [34].
Innate Immune Adjuvants	TLR Agonists (CpG, R848)	Activate TLR9 and TLR7/8 pathways in DCs and macrophages, promoting tumor antigen presentation and T-cell recruitment [44]. Clinical trials are evaluating liver-targeted delivery of these agonists.
Trained Immunity-Based Vaccines	BCG/β-Glucan + Tumor Antigen	A combination strategy to engage both innate and adaptive immunity. β-Glucan-based vaccines have shown enhanced T-cell responses in preclinical studies [45,46].
Combination Therapy	Checkpoint Inhibitor + Trained Immunity Inducer	Combining β-glucan or BCG with anti-PD-1 therapy increased immune infiltration and tumor control in preclinical models [2]. Atezolizumab + Bevacizumab already demonstrates microenvironment modification.

## Abbreviations

APC	Antigen-Presenting Cell.
BCG	Bacillus Calmette–Guérin
CAR	Chimeric Antigen Receptor
CTLA-4	Cytotoxic T-Lymphocyte-Associated protein 4
DC	Dendritic Cell
HBV	Hepatitis B Virus
HCC	Hepatocellular Carcinoma
HCV	Hepatitis C Virus
HLA	Human Leukocyte Antigen
ICI	Immune Checkpoint Inhibitor
IFN-γ	Interferon gamma
IL	Interleukin (e.g., IL-1β, IL-6, IL-10, IL-12, IL-15, IL-18)
iNOS	Inducible Nitric Oxide Synthase
LPS	Lipopolysaccharide
MDSC	Myeloid-Derived Suppressor Cell
MHC	Major Histocompatibility Complex
mTOR	Mammalian Target Of Rapamycin
NASH	Non-Alcoholic Steatohepatitis
NK	Natural Killer (cells)
NKG2A	Natural Killer Group 2A
NLRP3	NOD-Like Receptor Protein 3
NOD2	Nucleotide-binding Oligomerization Domain-containing protein 2
PD-1	Programmed cell Death protein 1
PD-L1	Programmed Death-Ligand 1
ROS	Reactive Oxygen Species
TAM	Tumor-Associated Macrophage
TAN	Tumor-Associated Neutrophil
TGF-β	Transforming Growth Factor beta
TLR	Toll-Like Receptor
TME	Tumor Microenvironment
